# "Harnessing the power of soil microbes: Their dual impact in integrated nutrient management and mediating climate stress for sustainable rice crop production" A systematic review

**DOI:** 10.1016/j.heliyon.2024.e41158

**Published:** 2024-12-12

**Authors:** Said H. Marzouk, Damiano R. Kwaslema, Mohd M. Omar, Said H. Mohamed

**Affiliations:** a Ministry of Education and vocational training, Zanzibar, Tanzania; b Department of Soil and Geological Science, Sokoine University of Agriculture, Tanzania; c Tanzania Agricultural Research Institute (TARI), Mlingano Center, Tanzania; d Department of Molecular Biology and Biotechnology, University of Dar-es-salaam, Tanzania

**Keywords:** Climate change, Plant-soil microbe interactions, Carbon sequestration, Food security, Integrated nutrients management, Soil health

## Abstract

Sustainable agricultural practices are essential to meet food demands for the increased population while minimizing the environmental impact. Considering rice as staple food for most of the world's population, it requires innovative approaches to ensure sustainable production. In this paper, we create a hypothesis that integrated nutrient management (INM) acts as a source of energy for microbes and improves the physical, chemical and biological properties of soils, but the current understanding of how soil microbiomes interact in integrated nutrient management toward mediating climate stress to support sustainable rice crop production is limited. Hence, we develop literature search through Preferred Reporting Items for Systematic Review and Meta-Analysis (PRISMA) to explore the hidden knowledge related to that question. The outcomes of the study are postulated as a viable option to minimize excessive chemical fertilizers and promote organic-based nutrient management that directly impacts microbial consortia. This review uncovered that plant-microbe interactions and nutrient transformation depend heavily on soil microbes while the abundance, diversity, and activity of soil microbiome is enhanced more with integrated nutrient management than with sole synthetic fertilizers. Through their ability to enhance nutrient availability and uptake, improve soil structure, heavy metal detoxification, salinity and drought tolerance, and suppress pathogens, they can alleviate abiotic stress associated with climate change. Therefore, optimization of microbial communities serves as a potential mechanism for INM to enhance rice yield and mitigate climate stress. This would improve soil health and enhance the resilience of the rice plant to climate change. However, despite various benefits obtained through INM and microbes in paddy production systems, the literature indicated that adoption of this technology is limited to smallholder farmers due to lack of knowledge, unavailability of sufficient organic materials and poor understanding of the long-term impacts associated with over-application of chemical fertilizers. Therefore, scientists must translate several research discoveries related to sustainable agriculture into simple language that can be adopted by farmers and future research should be a farmers-participatory approach to generate awareness investments and knowledge of farmers in adopting sustainability measures. Additionally, research could focus on identifying mechanisms by which microbiomes improve nutrient uptake and rice growth and how these mechanisms can be optimized through integrated nutrient management strategies with regard to climate stresses.

## 1 Introduction

Microorganisms such as bacteria, fungi, viruses, archaea, and protists make up soil ecosystems [[1], [2], [3]] and play a crucial role in agricultural ecosystem health and productivity [4,5]. They perform variety of functions, including nutrient cycling, organic matter decomposition, disease suppression, plant growth promotion and resistance to stressors, such as drought and heavy metal pollution [6,7]. Climate change can disrupt microbiomes and negatively impact changes in specific functions performed by soil microbes [6]. Additionally, agricultural practices such as conventional tillage and severe application of chemical fertilizers have been shown to affect soil microbial communities [8,9]. However, using certain agricultural practices such as integrated nutrient management (INM), crop rotation, cover cropping, and reduced tillage can promote microbial health and improve nutrient cycling [[10], [11], [12], [13]]. Similarly, using microbial inoculants and other biostimulants can help to restore microbiomes that have been damaged by climate. Therefore, by understanding effective ways of managing soil microbiomes, it is possible to mitigate the effects of climate stress on nutrient cycling and enhance soil health [6,14].

Soil microbial communities are reported to be affected by climate change in terms of abundance and function [15,16]. The study by Fu et al. [4] demonstrated that both extreme and low temperatures alter relative abundance of soil microorganisms. According to Ref. [17], soil microbes play a significant role in maintaining soil organic carbon stocks (SOC), but climate change is likely to affect future predicted effects of soil temperature on soil total nitrogen pools if warming exceeds 2 °C. It is therefore imperative to mitigate climate change for sustainable agriculture and to understand how climate change affects specific microbial communities as well as to monitor microbial adaptation to altered stresses [16].

One of the most important food crops in the world is rice, which depends heavily on efficient nutrient management and climate resilience. However, currently, paddy production systems have experienced severe chemical fertilization particularly nitrogenous fertilizer [18,19]. This trend with continuous climate changes has led to severe deterioration in soil fertility and health [20], thereby changing soil moisture availability, increasing disease and pest incidences [21,22], and posing a negative impact on rice crop production and environmental safety [15,23]. Furthermore, it was projected that by 2050 approximately 22 % of the world's important crops will be negatively affected by climate change [24], particularly rice crops [25]. Considering the negative impacts of climate change, global demand for land resources and needs to enhance agricultural production several agricultural conservation measures have been established [26,27] such as climate-smart agriculture [26,28], conservation agriculture [29,30], integrated nutrients management practices (INM) [31,32], agro-forestry [33], integrated use of soil microbes such as phosphate solubilizing microorganisms (PSB) and potassium solubilizing microorganism (KSB) [[34], [35], [36]] and regenerative agriculture. Overall, basic concepts of conservation measures aim to increase crop performances while maintaining soil fertility and curtail high doses of applying chemical fertilizers for long-term sustainable crop production [[37], [38], [39]].

Integrated nutrient management (INM) can be defined as optimum use of native sources of nutrients (locally available resources) such as organic manure, crop residues, biological N fixation (eg. legumes and *Azolla*), and chemical fertilizer in a balanced manner to increase nutrients recovery and minimizes loss [40,41]. Rice crop production remains better with INM; however, organic fertilizers are slow-release and may not be enough to meet plant requirements. This is the main reason apart from being labor-intensive for farmers to prefer more chemical fertilizers [42]. INM has been demonstrated to enhance soil organic carbon (SOC), improve nutrient use efficiency, microbial activities and diversity, growth and grain yield of rice, and long-term environmental conservation compared to other soil management practices [[43], [44], [45]]. This practice also aids in proper utilization of chemical fertilizers that minimize loss, boost soil quality and supply immediate nutrition needed to rice crops [41,46]. Yet, to date, rather contentious results have been obtained regarding impacts of INM on soil microbial diversity and their contributions towards mediating climate stress in paddy production systems. Hence this study reviews, summarizes and report in detail the results of modern published papers from 2000 to 2023 to establish baseline of hidden values of microbes in INM practices for mediating climate stress in Paddy production systems.

## 2 Data collection and compilation

**Fig. 1:**
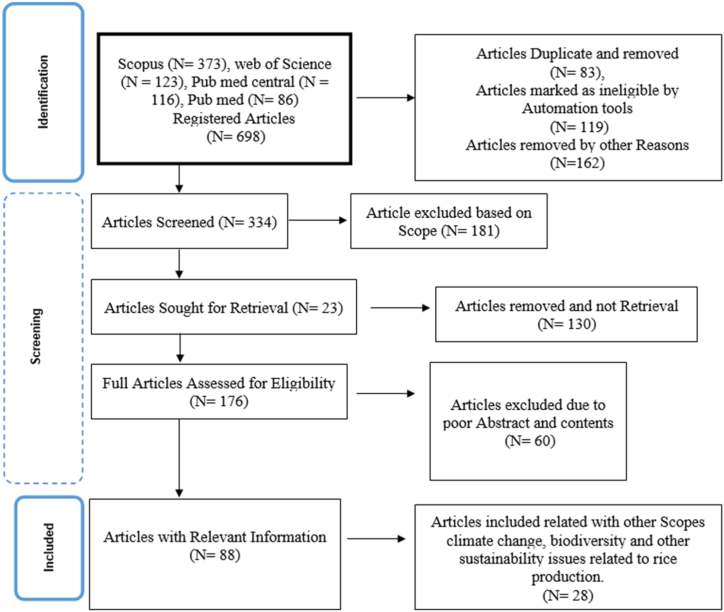
Representation of literature search based on the PRISMA flow diagram, modified from other researcher.

A systematic literature search was done through Preferred Reporting Items for Systematic Review and Meta-Analysis (PRISMA) followed by other researchers [39,47,48] from four databases Pub med, Scopus, PubMed Central, and Web of Science from January 2000 to January 2023 to outline impacts of microbes in INM practices for mediating climate stress in Paddy production systems using paper published in English language only (Fig. 1). Selection of years was done to obtain more recent publications with advanced methodology and statistical approaches. The search strings “soil microbes”, “climate stress”, “Integrated nutrient management” and “paddy” returned 670 articles. Articles with duplication and those that are out of scope were removed (Fig. 1). Articles included (116) were selected by checking abstracts and results that contained variables focusing on search titles, and valid experimental design and statistical analysis. We included results from any country related to scope.

## 3 Findings of the review

### 3.1 Benefits of integrated nutrient management practices (INM) on improving soil microbial health in paddy systems

Studies demonstrated that, Integrated Nutrient Management (INM) practices significantly enhance soil microbial health in paddy systems by combining organic and synthetic fertilizers, which provide diverse sources of organic carbon and nutrients. This approach improves soil physical properties, creating favorable conditions for microbial activity and diversity. INM increases microbial biomass carbon, nitrogen, and enzyme activities, which are crucial for nutrient cycling and soil health. For instance, research has proved that INM boosts microbial enzyme activities and enhances soil organic matter content, serving as a primary source of energy and nutrients for soil microbes [49,50]. Overall, INM practices lead to increased microbial populations and enzyme activities, particularly with the use of NPK + Azolla and NPK + FYM, thereby promoting a healthier and more productive soil ecosystem.

Paddy soils harbor diverse and active soil microbial communities that perform crucial functions such as nutrient cycling, organic matter decomposition, plant growth promotion, and disease suppression [50]. However, these soil microbial properties can be impaired by the intensive and continuous application of synthetic fertilizers, which can induce soil acidification, nutrient imbalance, organic matter depletion, and soil degradation [51,52]. To overcome these challenges, integrated nutrient management (INM), which combines organic manure and reduced synthetic fertilizer, has been advocated as a sustainable strategy to improve soil fertility and crop productivity in paddy systems [49,52,53]. This section highlights INM's contribution to improved microbiological indicators of soil health in paddy systems, including the associated mechanisms. The improvement of microbial properties is further viewed as a key mechanism by which INM enhances crop yields and mitigates climate stresses. Table 1 summarizes various research reports on beneficial effects of INM on soil microbial properties and associated mechanisms under paddy system. Most scientific studies on the matter have been conducted in Asian countries particularly India and China (Table 1), and less in Africa. Hence the transfer of this knowledge to other developing countries may be a good option to improve rice production and conserve soil fertility.

**Table 1:** Summary of the effects of Integrated Nutrient Management (INM) on soil microbial properties and underlying mechanisms in paddy systems.

Nutrient Management Practices	Duration	Effects of INM on Soil Microbial Properties	Mechanisms Underlying the Improvement of Soil Microbial Properties by INM	References
Synthetic fertilizers (NPKS), farmyard manure (FYM), and NPKS + FYM	34 years	INM increases microbial biomass carbon, microbial biomass nitrogen and enzyme activities (phosphatase and dehydrogenase, β-glucosidase, L-leucine aminopeptidase, N-acetyl-glucosaminidase, and β-cellobiosidase).	● The direct supply of organic substrates from INM increases the activity and diversity of phosphate-solubilizing bacteria, which produce alkaline phosphatase to release phosphorus from organic matter. ● Indirectly, balanced nutrient supply stimulates more plant growth and rhizodeposition, which provides more carbon substrates for soil microorganisms and increases their enzyme activities	[54]
Synthetic fertilizers (NPK), farmyard manure (FYM), and NPK + FYM	33 years	INM more significantly increased soil microbial enzyme activities (acid and alkaline phosphatase, dehydrogenase, cellulase and protease) than other treatments.	● Directly supply of organic substrates triggers increment in activities of soil microbial enzyme involved in N, P, and C cycling as evidenced by strong positive correlations between enzyme activities and soil organic carbon. ● Indirectly, INM provides favorable physicochemical conditions such as soil pH, lower bulk density (increased porosity), and nutrient availability which enhances microbial growth and activities.	[57]
Synthetic fertilizers (NPK), and NPK + Azolla or FYM or Rice stubble	32 years	INM using NPK + Azolla and NPK + FYM increased soil microbial population of bacteria and fungi by 76.8 % and 109.02 % respectively. Also increased microbial biomass carbon and soil enzyme activity.	● Stimulation of microbial enzymes in presence of organic substrates supplied by INM. ● NM improves the SOM content, a main source of energy and nutrients for soil microbes	[70]
Synthetic fertilizer (NPK), and NPK + FYM, green manure, wheat straw, or Sesbania aculeate	31 years	INM reduced soil bulk density with increasing infiltration rate, water retention capacity, SOC, and TN, compared to the sole application of synthetic fertilizers and control.	● NM improves the SOM content, a main source of energy and nutrients for soil microbes	[71]
Synthetic fertilizer (NPK), sole cattle slurry, and co-application of inorganic fertilizers with cattle slurry, household biowaste and biogas residues	25 years	The application of organic fertilizers positively enhances enzyme activities. However, the sole application of synthetic fertilizers and disturbed tillage did not show any significance in microbial communities.	● Directly supply of organic substrate through INM supplies source of carbon and energy for microbial growth and stimulates enzyme activities	[15]
Synthetic fertilizers (NPKSZn) + Poultry manure	33 years	INM increased soil bacteria and improved soil organic carbon stock by 27.98 %	● A positive correlation (r −0.94) was found between C content of mean weight diameter of water-stable aggregates and soil bacteria population, which provide evidence of vital contribution of soil bacteria for carbon sequestration	[72]
Synthetic fertilizer (NPK), and NPK + rice straw (NPKR) or Reduced NPK fertilizer and rice straw (L-NPKR)	21 years	Both NPKR and L-NPKR significantly shifted overall soil bacterial composition, enriching the beneficial microbial groups i.e. Bradyrhizobiaceae and Rhodospirillaceae families. Altered the soil microbial functional structure with increased diversity and abundances of genes for carbon and nitrogen cycling compared to unfertilized control or sole SF or sole rice straw	● Substrate availability triggers upregulation of genes encoding enzymes associated with N and C cycling. ● The increment in functional genes for N and C cycling is attributed to enhanced abundance of functional bacterial communities such as Bradyrhizobiaceae and Rhodospirillaceae families for N and increased C substrate from INM.	[73]
Synthetic NPK fertilizer, poultry manure, and vermicompost	1 year	Slight increases in organic and total carbon contents in soils were observed due to the addition of PM and VC at the rate of 5 t ha 1.	● NM improves the SOM content, a main source of energy and nutrients for soil microbes	[45]

INM can enhance soil microbial health by providing diverse sources of organic carbon and nutrients, improving soil physical properties, and creating favorable conditions for microbial activity and diversity. Several studies have reported the positive effects of INM on soil microbial properties in paddy soils compared to sole synthetic fertilizers application. For example, Zhang et al. [54] found that INM increases microbial biomass carbon, microbial biomass nitrogen and enzyme activities (phosphatase and dehydrogenase, β-glucosidase, L-leucine aminopeptidase, N-acetyl-glucosaminidase, and β-cellobiosidase). The mechanisms underlying the improvement of soil microbial properties by INM include the direct supply of organic substrates which increases the activity and diversity of beneficial bacteria and indirectly stimulates more plant growth and rhizodeposition which provides more carbon substrates for soil microorganisms [55,56]. Saha et al. [57] also found that INM increased soil microbial enzyme activities (acid and alkaline phosphatase, dehydrogenase, cellulase and protease) more significantly than other treatments. These improvements are due to the direct supply of organic substrates which triggers an increment in activities of soil microbial enzymes involved in N, P, and C cycling as well as indirectly providing favorable physicochemical conditions such as soil pH and nutrient availability which enhances microbial growth and activities [57].

In addition to the studies mentioned earlier, several other studies have reported the positive effects of INM on soil microbial properties in paddy soils. For example, Gogoi et al. [52] found that INM using NPK + Azolla and NPK + FYM increased soil microbial population of bacteria and fungi by 76.8 % and 109.02 % respectively, as well as increased microbial biomass carbon and soil enzyme activity. The mechanisms underlying these improvements include the stimulation of microbial enzymes in the presence of organic substrates supplied from INM and the improvement of soil organic matter (SOM) content, which is a main source of energy and nutrients for soil microbes [52]. Tang et al. [58] reported that INM improved soil microbial community structure, while Xue et al. [59] found that INM reduced soil bulk density with increasing infiltration rate, water retention capacity, SOC, and TN compared to the sole application of synthetic fertilizers and control. Chen et al. [15] found that the application of organic fertilizers positively enhances enzyme activities, while Ding et al. [60] reported that both recommended rate of NPK + rice straw (NPKR) and reduced NPK fertilizer and rice straw (L-NPKR) significantly shifted overall soil bacterial composition, enriching beneficial microbial groups such as Bradyrhizobiaceae and Rhodospirillaceae families and altering the soil microbial functional structure with increased diversity and abundances of genes for carbon and nitrogen cycling compared to unfertilized control or sole synthetic fertilizer or sole rice straw. These improvements are attributed to substrate availability triggering upregulation of genes encoding enzymes associated with N and C cycling as well as enhanced abundance of functional bacterial communities for N and increased C substrate from INM [60]. Lian et al. [61] found that long-term application of organic manure or organic manure plus chemical fertilizer (INM) increased the abundances of Acidobacteria_Gp6 and Planctomycete compared to other treatments, while Urmi et al. [45] observed slight increases in organic and total carbon contents in soils due to the addition of poultry manure and vermicompost at the rate of 5 t ha^−1^.

Interestingly, from biochemical point of view, Integrated Nutrient Management (INM) enhances soil microbial health in paddy systems by stimulating various biochemical processes [62,63]. The addition of organic and inorganic nutrients boosts microbial metabolism, leading to increased enzyme production, such as cellulases and phosphatases, which aid in nutrient cycling and organic matter decomposition [55,64]. This process improves soil structure through microbial exudates that enhance soil aggregation. Additionally, INM promotes beneficial symbiotic relationships, like mycorrhizal associations, which improve nutrient uptake, and antagonistic interactions that protect plants from pathogens [55,65]. Overall, INM activates a complex biochemical machinery that enhances soil fertility and plant health.

The physical and chemical properties of soil significantly influence soil microbial properties and the effectiveness of Integrated Nutrient Management (INM) in paddy systems [66]. Soil texture and structure affect microbial habitats and aeration, with fine-textured soils providing stable environments for microbes [67]. Soil pH and organic matter content are crucial, as they determine nutrient availability and microbial diversity. For instance, neutral to slightly acidic pH levels support diverse microbial communities, while high soil organic matter enhances microbial activity by providing essential carbon sources [68]. INM practices improve these soil properties by adding organic and inorganic nutrients, which enhance soil structure, nutrient cycling, and microbial health. This creates a favorable environment for microbes, leading to better soil fertility and crop productivity [62,69].

However, some research gaps still need to be addressed to better understand the mechanisms and interactions of INM on soil microbial health and crop productivity in paddy soils. For example, the effects of different types and sources of organic manures, such as green manure, biochar, compost, biowaste, biogas residues, etc., on soil microbial properties and their interactions with synthetic fertilizers are not well studied. The influence of INM on soil functional genes, such as those involved in nitrogen cycling, carbon cycling, and stress response, is also not well understood. Moreover, the long-term effects of INM on soil microbial health under different climatic conditions, such as drought, flooding, salinity, etc., are also not well explored. Despite the promising nature of INM in promoting native soil microbiome, the review reveals a lack of studies on using rhizobacteria that boost plant growth as inoculum along with INM to mitigate climate stress in rice fields. Additionally, there is a lack of studies on the effects of INM on soil microbial properties in paddy systems in regions outside of Asia, particularly in Africa. Therefore, more studies are needed to fill these knowledge gaps and provide more insights into how INM improves soil microbial health and crop yield in paddy soils compared to synthetic fertilizers alone.

### 3.2 Effect of soil microbial nutrient recycling, yield and productivity in paddy system under integrated nutrient management (INM) practices

This section of the review sheds light on soil microbes' crucial role in nutrient recycling in paddy systems and their contribution to mitigation of climate stresses by examining existing literature. Furthermore, the review highlights the significance of harnessing the potential of soil microbes to enhance agricultural practices and promote sustainable paddy cultivation. The results provide valuable insights for researchers, policymakers, and farmers, guiding more informed decisions and innovative approaches to optimize nutrient utilization and improve overall productivity in paddy fields. Details of numerous research reports on soil microbial benefits under INM practices in rice crop production are summarized in Table 2. The studies summarized in this section employed several common research methodologies to evaluate the effects of INM practices on soil microbial nutrient recycling, yield, and productivity in paddy systems. These methodologies include field experiments, soil sampling and analysis and crop yield measurements. The nutrient management practices are categorized into organic and inorganic fertilizers. Among these practices, the combination of organic and inorganic fertilizers, often referred to as Integrated Nutrient Management (INM), is the most commonly used and has shown significant effects on soil health and crop productivity. Organic fertilizers, such as compost, farmyard manure, and green manure, provide a slow-release source of nutrients and improve soil organic matter content. They enhance soil structure, water retention, and microbial activity, creating a favorable environment for plant growth. Sole synthetic fertilizers such as urea, diammonium phosphate (DAP), and potassium chloride, offer an immediate supply of essential nutrients. They are crucial for meeting the nutrient demands of high-yielding rice varieties, especially during critical growth stages. The combination of organic and inorganic fertilizers in INM practices leverages the benefits of both types of fertilizers. Organic fertilizers improve soil health and microbial activity, while inorganic fertilizers provide the necessary nutrients for optimal crop growth. Studies have shown that INM practices significantly increase rice grain yield, nutrient uptake, and use efficiencies compared to the sole application of either organic or inorganic fertilizers.

**Table 2:** Summary of the mechanisms underlying microorganisms' dual role in rice yield and climate stress relief under INM.

Summary of the mechanism underlying microorganisms under INM	Nutrient management practices (Organic and inorganic fertilizers used)	Impact on Rice Yield under INM	Direct and indirect response in Alleviation of Climate Stress under INM	References
Under INM Microorganisms facilitate decomposition of organic matter, leading to better nutrient cycling in the soil.	Recommended inorganic fertilizers, poultry manure and vermicompost.	Combined application of organic and inorganic fertilizers resulted in higher rice grain yield, nutrient uptake and use efficiencies, which were satisfactory compared to the control.	Improved microbes enhance availability of essential nutrients to plants and improve their resistance to climate stressors	[45]
Under INM abundance of Nitrogen-fixing bacteria (e.g., Rhizobium and Azotobacter) and cyanobacteria increases, which can convert atmospheric nitrogen into a useable form (ammonia or nitrates) that rice plants can utilize	Azolla, synthetic N fertilizer.	Compared to the full N rate, reduced N with Azolla increases N recovery by 46.5 and 39.1 %, and reduces NH3 volatilization by 52.3 and 64.3 % without decreasing grain yield.	This process reduces the reliance on synthetic nitrogen fertilizers, reduces costs, and minimizes environmental pollution.	[115]
Under INM Certain microorganisms play an important role in reducing greenhouse gas emissions, such as methane and nitrous oxide, from paddy fields.	Azolla green manure, Poultry-litter biochar and inorganic fertilizers.	Co- application of Azolla and biochar offers a novel approach to increasing rice grain yield and minimizing emissions. Additionally, combining Azolla and biochar significantly increased rice grain yield by 27.3%–75.0 %.	This helps mitigate the contribution of rice cultivation to global warming and improve nutrient use efficiency	[95]
Under INM certain microorganisms help plants in water-stressed conditions by improving their water use efficiency	Biofertilizers and synthetic fertilizers	The use of biofertilizers integrated with chemical fertilizers significantly increases the number of tillers, plant height, economic benefit, and grain yield compared to chemical fertilizers alone.	They can assist in the synthesis of stress-responsive compounds and promote deeper root systems, allowing rice plants to access water from deeper soil layers.	[116]
Under INM Microorganisms facilitate the decomposition of organic matter, leading to better nutrient cycling in the soil	Applying organic manure with good agricultural practices (GAP).	The average grain yields significantly increased by 1 t ha−1 in 2013 and 2.7 t ha−1 in 2014 when farmers practiced good agricultural practices compared to farmers' management practices.	This enhances the availability of essential nutrients to plants, improving their resistance to climate stressors.	[117]
Under INM microbial activities contribute to soil aggregation and the formation of stable aggregates and Beneficial microorganisms can act as natural biocontrol agents against plant pathogens, reducing disease incidence and severity	Azolla, mucuna green manure, and inorganic fertilizers.	Application of mucuna with N fertilizer significant (P < 0.05) increases 0.8 t ha−1 grain yield compared to control. Azolla incorporation increases grain yield by 1.4 t ha−1 which is not a significant difference with full N fertilizer alone.	This improves soil structure, leading to better water infiltration and retention, reduced soil erosion, and increased drought tolerance of rice plants. Additionally, healthy plants are better equipped to cope with climate stresses.	[118]
Under INM Nitrogen-fixing microorganisms contribute to nitrogen availability in the soil and reduce the need for synthetic nitrogen fertilizers with great potential in reducing methane and other greenhouse gas.	Azolla with a recommended dose of N	The highest grain yield of 3.95 t/ha with the highest straw and grain uptake of 75.4 kg N ha-1, 32.7 kg P ha-1, and 8.5 kg S ha-1 were recorded in the Azolla plot plus 40 kg N through urea.	This, in turn, leads to lower greenhouse gas emissions and less energy consumption during fertilizer production and certain microorganisms can competitively exclude or suppress methane-producing bacteria, reducing methane emissions from flooded fields.	[79]
Under INM certain microorganisms produce plant growth-promoting substances such as phytohormones (e.g., auxins, gibberellins) and siderophores	Organic matter	The yield of rice and wheat, microbial biomass carbon (MBC) and carbon stocks were significantly improved under organic matter application compared to full nitrogen dose. However, SOC stock loss in rice was higher than that in wheat.	These substances stimulate root growth, improve nutrient uptake, and enhance the plant's ability to withstand biotic and abiotic stresses, resulting in increased rice yield.	[32]
Under INM certain microorganisms, like phosphate-solubilizing bacteria and fungi, can release phosphorus from insoluble forms in the soil, making it available to rice plants.	Phosphate rocks, organic matter, and synthetic fertilizers	Applying phosphate rocks (eg. Minjingu) is an alternative method to minimize P deficiency in East Africa. Additionally, organic matter supplies N in soils and increases crop yields.	This enhances phosphorus uptake and promotes better root development and overall plant growth.	[46]
Under INM Some microorganisms act as biocontrol agents, controlling pests and diseases that can negatively impact rice yield.	Rice straw, burnt rice husk and legume residue	The highest grain yield of 3.40 t/ha was recorded in integrated nutrient application with burnt rice husk and legume residue compared to the soil with no residue treatments.	certain entomopathogenic fungi can infect and kill insect pests, reducing crop damage and promoting healthier plant growth.	[91]
Under INM Microorganisms, especially bacteria and fungi, decompose organic matter present in the soil and convert complex organic compounds into simpler mineral forms, releasing essential nutrients such as nitrogen, phosphorus, and potassium.	NPK and farm yard manure	The application of NPK + FYM significantly increased the soil labile carbon, and water-soluble carbon; and reduces bulk density over control.	This process, known as mineralization, makes these nutrients readily available for plant uptake, thus supporting rice growth and yield	[43]
Under INM amendment microbial species richness which are often involved in degradation of complex organic compounds increased	NPKS, composted cattle manure, composted swine manure	Application of composted cattle manure improve soil fertility and crop yield in a submerged rice cropping system.	This process improves soil structure, Organic carbon and increase rice yields in submerged environment	[119]
Under INM Microbial activities contribute to soil aggregation and the formation of stable aggregates.	Recommended rates of NPK, poultry manure, cow dung, and rice straw	Application of cow dung, poultry manure, and rice straw resulted in 36, 28 and 37 % loss of applied C through emission and 10, 30 and 49 % sequestration, respectively.	This improves soil structure, leading to better water infiltration and retention, reduced soil erosion, and increased drought tolerance of rice plants.	[120]
Under INM and rotation with legumes, population of N-fixing microbes was increased and enhanced biological fixation and improved soil quality.	50 % of chemical fertilizers, legume crop (Vigna radiate) green manure, farmyard manure, wheat stubble.	The use of INM significantly increases net nitrogen mineralization and improves NUE. Also enhances NH4+ availability and uptake by rice plants in highly reduced conditions. Finally increases SOC sequestration and subsequently maintains higher grain yield.	Improved nutrient cycling and minimizes the need for chemical fertilization. Increased NUE reduces N emission through volatilization and denitrification.	[42]

**Fig. 2:**
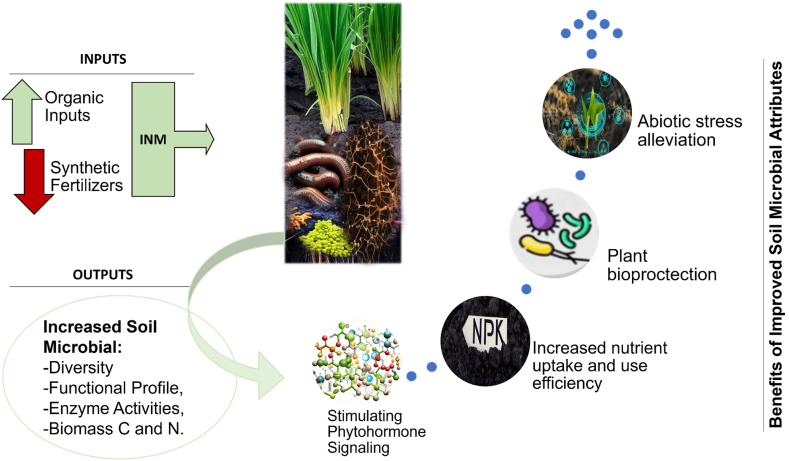
Illustration showing a conceptualized contribution of improved soil microbial health attributes under INM.

Under INM soil microbes play an important role in influencing plant productivity, mediating effects of climate and improving soil health [3,74,75]. The literature explored the impact of synthetic chemical fertilization on rice yields [54,[76], [77], [78]] but also revealed the adverse effects of intensive chemical use and extreme climatic factors on soil quality, fertility, biodiversity, and agricultural sustainability [2,79]. Various ecological stresses, including diseases eg. Rice Blast (*Magnaportheoryzae*), Bacterial Leaf Blight (*Xanthomonas oryzaepv. oryzae*), Sheath Blight (*Rhizoctonia solani*), Brown Spot (*Bipolarisoryzae*), Rice Yellow Mottle Virus (RYMV), and Bakanae Disease (*Gibberellafujikuroi*) [37,80], pests, weeds, drought, and salinity [[80], [81], [82]], have been identified as challenges in rice crop production systems. However, the study also acknowledges the crucial role of soil microbes in nutrient cycling, disease suppression, and stress tolerance. Integrated Nutrient Management (INM) practices have shown promise in harnessing the power of these microbes, leading to improved nutrient availability, plant health, and stress resilience, resulting in increased sustainable rice yields and food security [74,75]. Fig. 2 shows conceptualized contribution of improved soil microbial health attributes under integrated nutrient management.

The review further highlights the importance of organic and inorganic sources of fertilizers and the role of soil microbes in improving nutrient availability. Several organic fertilizers such as cow manure, Azolla compost, rice straw compost, rice straw biochar, burnt rice husk, legumes, vermicompost, and inorganic fertilizers such as urea, diammonium phosphate (DAP), calcium nitrate (CAN) were applied at different rates in rice crop production [[83], [84], [85]]. INM practices were found as an effective tool for improving microbial population that directly increasing nutrient use efficiency, enhancing crop performance, and mitigating ecological stresses like salinity and heavy metal pollution. Studies have demonstrated the positive impact of INM on nutrient supply capacity, microbial diversity, and enzyme activities in paddy soils, contributing to higher photo-assimilates and dry matter accumulation [2,32,38,40,86,87].

For example [88], performed two-year field experiments on the sole application of Azolla with reduced N-rate and observed that reduced N alone significantly decreases NH_3_-volatilization without an increase in grain yield compared to the full N rate, however, reduced N substituted with Azolla significantly decreased NH_3_-volatilization by 59.72 % thereby decreasing floodwater pH and temperature and significantly increasing nitrogen use efficiency by 23.73 % compared to control without reduction in grain yield. The grain yield increase due to the application of reduced N with different organic fertilizers was reported. For example, reducing N with velvet mucuna increases grain yield by 0.8 t ha^−1^ compared to control [89], cattle manure [90], burnt rice straw [91] and farm yard manure [92]. In long-term bases [93], observed that sole application of farm yard manure (FYM) and green manures (e.g. Lablab, cowpea and Stylosanthes guianensis) increase rice grain yields from 0.7 to 3.1 t ha^−1^ over control with more preservative effect when the two (farm yard manure and green manures) were combined. This review observed that different recommended rates of both organic and inorganic fertilizers were reported in different parts of the world (Table 2) and overall literature search indicated that the global price of inorganic fertilizers is increasing and becoming unaffordable for resource-poor smallholder farmers, particularly in Sub-Saharan Africa. Therefore, identifying the optimum dose of integrated nutrient application and soil microbes is required for an adequate supply of nutrients [94].

Another study by Ref. [95] observed that the co-application of *Azolla* and biochar offers a novel approach to increasing N and minimizing emissions in a rice production system, additionally, combining *Azolla* and biochar significantly increased rice grain yield by 27.3–75.0 %. The review further identified that INM enhanced the nutrient supply capacity of paddy soils thereby providing favorable environments to beneficiary microorganisms that favor vegetative growth resulting in higher photo-assimilates and dry matter accumulation [84,96].

Studies indicated that soil microbes improve availability of nutrients in the soil through various mechanisms such as mycorrhizal fungi that form extensive fungal hyphae with rice roots and enhance rice plant access to nutrients. Availability of essential nutrients contributes directly to rice yield. According to the findings of this review, phosphorus plays an important role in growth and development of rice crops [96,97], but its availability in paddy soils is often limited and become one of the most identified yield-limiting nutrients in lowland rice production systems over the world [46,98,99]. The study conducted by Ref. [100] points out some complicated chemistry in paddy production systems. In waterlogged conditions, the redox potential and soil pH decreased. This increases the solubility of iron (Fe^3+^to Fe^2+^) and manganese (Mn^4+^to Mn^2+^) and finally enhances P fixation [101]. However, being part of the problem, INM and the use of beneficiary microorganisms is also part of the solution to P deficiency in paddy production systems [35,102]. It was observed that the use of INM can significantly improve phosphorus use efficiency in paddy. For example, a study by Refs. [103,104] found that application of organic manures, such as farmyard manure, Azolla and vermicompost, along with chemical fertilizers, increased phosphorus use efficiency by 33–46 % compared to use of chemical fertilizers alone. Additionally, variety of soil microbes such as Azotobacter, Achromobacter, Rhizobium, Bacillus, Flavobacterium, Pseudomonas and Enterobacter dissolve fixed-P and other bacteria like acidophilic taxa can oxidize Fe (II) to Fe (III) and quickly hydrolyzes and precipitates as Fe (III)-hydroxides. These microbes were termed Phosphate Solubilizing Bacteria (PSB) and have direct impact on increasing P use efficiency, minimizing iron toxicity in anaerobic conditions and improving crop yields [105]. Therefore, INM and phosphate solubilizing microorganisms may be a good option to increase P use efficiency in paddy production systems.

Potassium on the other hand is one of the three primary macronutrients required for plant growth and development its deficiency can adversely affect crop yield and quality [106]. like phosphorus, availability of potassium in soil is often limited. However, the use of organic sources such as farmyard manure and compost (INM) can provide a slow-release supply of potassium to the soil and improve its availability to rice plants leading to improved crop yields [64]. Additionally, use of biofertilizers, such as nitrogen-fixing bacteria and mycorrhizal fungi enhances uptake of potassium by plants. For example, a study by (Walia et al., 2010) and Yuan et al. [64] found that application of organic manures, such as pig manure and rice straw, along with chemical fertilizers, increased potassium use efficiency by 16–27 %, micronutrient contents (Zn, Cu, Fe, and Mn) and rice yield from 8.54 to 9.56 t ha^−1^compared to sole chemical fertilizers. Additionally, several bacteria species such as Bacillus, Arthrobacter, Paenibacillus, Acidithiobacillus, Pseudomonas and Enterobacter dissolve potassium from insoluble K-bearing minerals such as muscovite, orthoclase, feldspar, illite and biotite, into soil solution and enhance seed germination, vegetative growth and control water-use efficiency [64,107,108]. Although INM can be an effective strategy to improve potassium use efficiency, optimal combination of organic and inorganic sources of nutrients depends on several factors, therefore researchers should develop customized INM plans for proper supply of nutrients.

The current review discovered that Sulfur (S) is an essential secondary nutrient for plant growth and development, and its deficiency can lead to reduced rice crop yields and quality [42]. However, unlike N-P-K fertilizers, in most cases, S is excluded in balanced nutrition in paddy production. The use of Integrated Nutrient Management (INM) can improve soil organic matter content, which can increase availability of sulfur to plants. Inorganic sources of sulfur, such as sulfate-containing fertilizers, can also be used to supplement soil's sulfur supply [45]. Additionally, use of soil microbes such as bacteria and fungi, also play an essential role in sulfur cycling and certain microbes, such as sulfur-oxidizing bacteria, can convert elemental sulfur to sulfate, which can be more easily taken up by rice plants. Other microbes, such as mycorrhizal fungi, can form symbiotic relationships with plants and enhance sulfur uptake [109,110].

Micronutrients (iron, zinc, copper, manganese, and boron) at a concentration of less than 0.5 g/kg of plant dry matter are also essential for plant growth and development and their deficiency restricts productivity, sustainability and stability of soils and induce other physiological disorders to plants [111,112] assessed 25 years of continuous application of synthetic and organic fertilizers and observed that application of green manures along with inorganic fertilizers increases concentration of micronutrients in soil and enhances plant uptake. Several studies have also investigated the effects of integrated nutrient management (INM) and microbial inoculants on uptake of micronutrients that also result in higher rice grain yield [2,57,113,114]. Overall, the review underscores the significant role of soil microbes in nutrient cycling and their positive impact on yield and productivity in paddy systems. The integration of microbial-based INM practices provides a promising avenue for sustainable rice production and soil health conservation. Understanding the intricate relationship between soil microbes and nutrient availability is crucial for optimizing agricultural techniques and ensuring long-term food security.

### 3.3 Role of microbes and microbe-induced INM on climate stress mitigation in paddy production system

Understanding the effects of INM on soil microbial diversity and function under various climate scenarios is crucial for predicting and managing the consequences of climate change on paddy systems. Climate changes are projected to have global impacts, leading to shifts in regional temperatures and altered precipitation frequencies [24,121]. Among various sectors, agriculture is expected to be highly affected [25]. In this review, we explored strategies for climate mitigation, with a focus on minimizing greenhouse gas (GHG) emissions through carbon sequestration [121]. We found a strong relationship between nutrient sources, Integrated Nutrient Management (INM), and soil microbes. Microbiomes were found to enhance nutrient uptake and improve plant growth, resulting in increased resilience to environmental stressors like drought, heat, and salinity, leading to better rice crop yields [122]. We view INM as a valuable tool to enrich soil microbiomes, enhancing the resilience of paddy production systems to climate stresses and ensuring food security.

Furthermore, our findings revealed that INM enhances soil fertility and health, leading to reduced reliance on chemical fertilization and subsequently minimizing GHG emissions from industrial fertilizer production. INM also contributes to carbon sequestration in the rice ecosystem [42]. Soil microbiomes play a critical role in cycling toxic heavy metals and gases, including methane, ammonia, and nitrous oxide, thereby contributing to GHG reduction [6]. Leveraging the interactions between soil microbes, plants, and the environment offers a promising approach to simultaneously enhance nutrient management and mitigate climate stress for sustainable rice crop production.

Soil microbes also exhibit responses to extreme climate events like droughts and floods, which are expected to become more frequent and severe due to climate change [123,124]. INM practices can enhance the diversity and function of soil microbes by providing balanced nutrient supply, organic matter, and beneficial microorganisms, leading to improved soil quality and plant health [125]. These practices increase the abundance and activity of nitrogen-fixing bacteria, phosphate-solubilizing bacteria and fungi, arbuscular mycorrhizal fungi (AM), and plant growth-promoting rhizobacteria, which help plants cope with climate stress regimes [32]. Additionally, INM can influence the sensitivity of soil microbes to extreme events and their contribution to greenhouse gas fluxes from paddy soils under climate change conditions [124]. Overall, the role of microbes and microbe-induced INM holds promise in mitigating climate stress and promoting sustainable rice production in the face of climate change challenges.

### 3.4 Some reported constraints that hinder the adoption of INM

The literature search identified that the use of Integrated Nutrient Management (INM) and Soil Beneficial Microbes can be hindered by several factors such as the unavailability of organic sources of nutrients eg. Farm yard manure, Azolla, and biofertilizers [30,39], lack of knowledge among farmers on the preparation and cycling of organic waste to produce quality compost [126] and the cost of INM and soil beneficial microbes can be high, which may make it difficult for farmers to afford [127]. Additionally, Using INM and soil-beneficial microbes can be time-consuming, as farmers need to be meticulous in their application and maintenance [42].

### 3.5 Conclusion and a way forward

This review has synthesized the current knowledge and evidence on the effects of integrated nutrient management (INM) and soil microbes on soil health, crop productivity, and climate resilience in paddy systems. The review has demonstrated that INM can provide multiple benefits for soil fertility, microbial activity, diversity, and function, as well as mitigate the negative impacts of excessive synthetic fertilizers and climate stress on soil and plant health. The review highlights the pivotal role of soil microbes in integrated nutrient management, where they significantly contribute to elevating rice yield and mitigating climate stress. These soil microbes actively enhance nutrient availability, stimulate plant growth, and facilitate adaptive responses to environmental stressors. Additionally, their involvement in regulating soil carbon cycling establishes a vital feedback loop to address climate change impacts effectively. Studies demonstrated that Long-term Integrated Nutrient Management (INM) practices, typically spanning several years, focus on enhancing soil microbial diversity, improving soil organic matter, and increasing nutrient use efficiency through sustained organic substrate provision, leading to long-term soil health and crop yield improvements. In contrast, short-term INM, usually lasting for a few years, aims to quickly boost microbial biomass and enzyme activities by providing a rapid supply of organic substrates, which enhances microbial activity and nutrient cycling in the short term. While both approaches aim to improve soil health and crop productivity, long-term INM offers more sustained benefits compared to the immediate but temporary effects of short-term INM. The review has identified some knowledge gaps and challenges that need to be addressed to optimize the potential of INM and soil microbes for sustainable rice production. Based on the findings of the review, we recommend the following actions for policymakers, researchers, and farmers.

i. Limited research exists to substantiate the influence of soil microbial properties on rice performance and climate mitigation within the context of integrated nutrient management practices, particularly in soils facing diverse abiotic stresses such as salinity, drought, and metal contamination, therefore more studies are recommended.
ii. Promote the adoption of INM practices that are suitable for different agroecological zones, soil types, and cropping systems, considering the local socio-economic and environmental conditions.
iii. Advocate research on development and dissemination of microbial inoculants and biofertilizers that can enhance the diversity and function of soil microbial communities and their beneficial effects on plant growth and stress tolerance.
iv. Monitor and evaluate the impacts of INM and soil microbes on soil quality, plant health, crop yield, GHG emissions, and carbon sequestration using standardized methods and indicators.
iv. Enhance the awareness and capacity of farmers, extension agents, and policymakers on the importance and benefits of INM and soil microbes for sustainable rice production and climate resilience.
v. Foster collaboration and knowledge sharing among stakeholders from different sectors and disciplines to facilitate the adoption and scaling up of INM and soil microbial technologies.

## CRediT authorship contribution statement

**Said H. Marzouk:** Writing – review & editing, Validation, Software, Methodology, Conceptualization. **Damiano R. Kwaslema:** Writing – review & editing, Methodology, Conceptualization. **Mohd M. Omar:** Writing – review & editing, Conceptualization. **Said H. Mohamed:** Writing – review & editing, Conceptualization.

## Declaration of competing interest

The authors declare that they have no known competing financial interests or personal relationships that could have appeared to influence the work reported in this paper.
